# Mutation of *hilD* in a *Salmonella* Derby lineage linked to swine adaptation and reduced risk to human health

**DOI:** 10.1038/s41598-020-78443-7

**Published:** 2020-12-09

**Authors:** Martina Tambassi, Melissa Berni, Chiara Bracchi, Erika Scaltriti, Marina Morganti, Luca Bolzoni, Jennifer R. Tanner, Gaëtan Thilliez, Robert A. Kingsley, Stefano Pongolini, Gabriele Casadei

**Affiliations:** 1 Risk Analysis and Genomic Epidemiology Unit, Istituto Zooprofilattico Sperimentale della Lombardia e dell’Emilia Romagna, Parma, Italy; 2 Quadram Institute Bioscience, Norwich Research Park, Colney, Norwich, UK; 3 University of East Anglia, Norwich, UK; 4 Present Address: National Microbioloby Laboratory, Public Health Agency of Canada, Winnipeg, MB Canada; 5 Present Address: Regional Epidemiology Unit, Istituto Zooprofilattico Sperimentale della Lombardia e dell’Emilia Romagna, Bologna, Italy

**Keywords:** Bacteria, Bacterial pathogenesis, Infectious-disease epidemiology

## Abstract

*Salmonella enterica* variants exhibit diverse host adaptation, outcome of infection, and associated risk to food safety. Analysis of the distribution of *Salmonella enterica* serovar Derby (*S*. Derby) subtypes in human and swine identified isolates with a distinct PFGE profile that were significantly under-represented in human infections, consistent with further host adaptation to swine. Here we show that isolates with this PFGE profile form a distinct phylogenetic sub-clade within *S*. Derby and exhibit a profound reduction in invasion of human epithelial cells, and a relatively small reduction in swine epithelial cells. A single missense mutation in *hilD*, that encodes the master-regulator of the *Salmonella* Pathogenicity Island 1 (SPI-1), was present in the adapted lineage. The missense mutation resulted in a loss of function of HilD that accounted for reduced invasion in human epithelial cells. The relatively small impact of the mutation on interaction with swine cells was consistent with an alternative mechanism of invasion in this pathogen-host combination.

## Introduction

*Salmonella* serovars differ in their adaptation to host species, a characteristic that affects their risk to human health^1^. Host adaptation is the ability of a serovar to circulate and persist in the population of a particular host species and may also be associated with distinct outcomes of infection. *Salmonella* serovars may be described as: (1) “specialists”, if exclusively associated with one specific host species; (2) “host adapted”, when commonly found in one particular host species but also detected in others and (3) “generalists”, when they infect multiple unrelated hosts^2^. Different host ranges exist even among lineages within the same serovar^3^. For example, *S*. Typhimurium is a monophyletic serovar that is predominantly generalist. However, specific lineages evolved to become host adapted to a variety of wild avian species, accompanied by changes in pathogenicity and risk to food safety^4,5^. *Salmonella* Derby is a polyphyletic serovar with three phylogroups adapted to swine, typically sequence types 40, 39 and 682 (ST40, ST39 and ST682), and one to poultry (ST71)^6^. In the European Union *S*. Derby is the most prevalent serovar in swine (53.3%), while the prevalence in turkeys is considerably lower (9.8%)^7^. Consistently, in the United States *S*. Derby was recently reported as the third most prevalent serovar in swine^8^. *S*. Derby ST39/ST40 are considered to be host adapted to swine^6^. However, *S*. Derby is also pathogenic for humans, being the fifth most commonly isolated serovar in the European Union^7^ and being reported in the United States among the agents of human salmonellosis^9^. Host adaptation has been correlated with genetic events such as generation of allelic variants by point mutation, genome degradation and horizontal gene transfer^1^. For instance, functional analyses of host-associated non-synonymous single nucleotide polymorphisms (nsSNPs) in *fimH*, encoding for the type 1 fimbrial adhesin, proved the role of allelic variation in host-specific adherence of *Salmonella *in vitro^10^. In *S*. Typhi, the causative agent of typhoid fever and a host specialist in humans, more than 4% of coding sequences are interrupted, resulting in the formation of pseudogenes, compared to only 0.9% of the generalist *S.* Typhimurium^11^. By contrast, *S*. Typhi horizontally acquired the *Salmonella* Pathogenicity Island (SPI)-7, a key factor for its pathogenesis^1^. In *S*. Derby, SPI-23 plays a role in infection of swine epithelial cells and is only present in the swine-adapted lineages ST40 and ST39^12^.

We investigated a specialized case of host adaption of a *S*. Derby ST40 lineage that has resulted in further adaptation to the swine host and decreased risk to human infection. Analysis of our database of *S.* Derby surveillance (446 isolates from 2012 to 2017), identified a specific PFGE profile that was significantly less likely to be isolated from human infections than from swine compared to isolates of other PFGE profiles of *S.* Derby. This swine adapted type showed altered interactions with swine and human epithelial cells in vitro, compared to isolates with closely related PFGE profiles. Here we report the genetic basis of the observed differential phenotype. We found that a single amino acid residue substitution in HilD, the master-regulator of SPI-1^13^, characteristic of the swine adapted PFGE profile, is responsible for the altered interaction with host cells.

## Results

### *Salmonella* Derby SXB_BS.0204 PFGE profile circulates in pigs but rarely infects humans

All *Salmonella* isolates of clinical, animal, food and environmental origin were serotyped and typed by PFGE within the joint human-animal surveillance of Emilia-Romagna Region of Italy. A total of 6335 isolates from sporadic cases of infection were received between July 2012 and February 2017. These were from human (4373 isolates), swine (1164 isolates), poultry (624 isolates) and bovine (174 isolates) sources. *S*. Derby accounted for 27.06% of swine isolates and only 3.45%, 2.74% and 0.80% of cattle, human and poultry isolates respectively, constituting, in our regional epidemiological context, a swine associated serovar, in line with already reported data^6–8^ (Supplementary Table 1). A total of 101 different PFGE profiles of *S*. Derby were identified (Supplementary Fig. 1) some of which varied in their prevalence in human compared with swine, as indicated by selection ratio (SR). The SR is defined as the ratio between the number of human isolates of a specific PFGE type over all human isolates of *S*. Derby and the number of swine isolates of the same type over all swine isolates of *S*. Derby. If SR = 1 the fraction of human isolates with that specific PFGE type equals the fraction of swine isolates with the same type. Correspondingly, a SR > 1 means that the fraction of human isolates with that specific PFGE type is greater than the fraction of swine isolates with the same type, whereas a SR < 1 indicates that the fraction of human isolates with that PFGE type is smaller than that of swine isolates with the same type. The second most abundant PFGE type in swine, SXB_BS.0204, was considerably less frequently isolated from humans than swine, with a SR = 0.0875 (*p*-value < 0.001). Conversely, isolates belonging to all other PFGE types had an SR close to 1, indicating that their prevalence in the human and swine compartments of isolates was similar. Therefore, SXB_BS.0204 exhibited a distinct epidemiology, characterized by abundance in swine but relatively low frequency of isolation from human infections.

### *Salmonella* Derby SXB_BS.0204 isolates have altered interaction with human and swine epithelial cells

**Figure 1:**
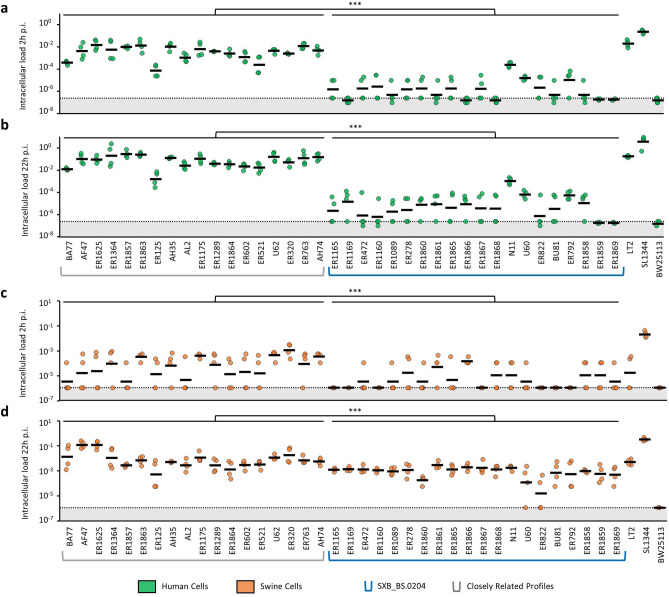
Virulence phenotype of *S*. Derby in human and swine cells. Results of intracellular loads at 2 h and 22 h post infection (p.i.) of *S*. Derby SXB_BS.0204 and closely related PFGE types in human cells (**a**,**b**) and swine cells (**c**,**d**). Results are expressed as intracellular bacteria recovered after 2 h and 22 h of infection divided by the inoculum. Two biological replicates were performed and each one was tested in duplicate. Dots represent the values of the replicates. Arithmetic means are indicated by horizontal lines. p-values from two-tailed Student’s t-test are reported (***p < 0.001). The dotted line corresponds to the limit of detection.

To evaluate if the epidemiology of isolates with the SXB_BS.0204 PFGE profile was associated with altered interaction with host cells, compared with *S*. Derby isolates with different PFGE profiles, we performed cell culture infection assays using human-derived INT-407 and swine-derived IPEC-J2 intestinal epithelium cell lines. A total of 20 isolates belonging to SXB_BS.0204 and 18 isolates with PFGE profiles closely related to SXB_BS.0204 (Closely Related Profiles, CRP) were compared (Supplementary Table 2). CRPs were identified as the PFGE types having more than 80% similarity with SXB_BS.0204 using the distance analysis method defined as Unweighted Pair Group Method with Arithmetic Average (UPGMA)^14^. The rationale of comparing the swine adapted SXB_BS.0204 isolates to closely related but non-adapted isolates was to keep the genetic differences between the two groups to a minimum, in order to facilitate the identification of the genetic determinants responsible for phenotypic differences. We measured the intracellular load of each isolate at 2 h post infection (p.i.) to determine the invasion rate. As after 2 h of infection we could not detect any intracellular bacteria in several infection replicates, especially for SXB_BS.0204 isolates because of their limited ability to invade cells, we also measured the intracellular load at 22 h p.i. to allow Salmonellae to multiply and thus to reach intracellular loads detectable under our experimental conditions. The largest differences between the two groups of isolates were observed in human cells (Fig. 1a,b) where the intracellular loads at 2 h and 22 h p.i. of SXB_BS.0204 isolates were respectively 3 and 4 logs lower (2 h p.i. = 1.19 × 10^–6^, 22 h p.i. = 4.41 × 10^–6^) than CRP isolates (2 h p.i. = 1.95 × 10^–3^, 22 h p.i. = 3.66 × 10^–2^, *p* < 0.001). An exception was SXB_BS.0204 isolate N11, that invaded human cells more than the other SXB_BS.0204 (intracellular load at 2 h p.i. = 2.47 × 10^–4^, intracellular load at 22 h p.i. = 1.04 × 10^–3^, *p* < 0.05). In swine cells, the intracellular loads at 2 h and 22 h p.i. of SXB_BS.0204 isolates (2 h p.i. = 4.55 × 10^–6^, 22 h p.i. = 7.39 × 10^–4^) were also significantly lower than those of CRP isolates (2 h p.i. = 3.91 × 10^–5^, 22 h p.i. = 5.77 × 10^–3^), but the difference was less than 1 log (Fig. 1c,d). Intracellular loads at 2 h p.i. (1.14 × 10^–5^) and at 22 h p.i. (1.77 × 10^–3^) of N11 were not significantly different from those of the other SXB_BS.0204. Based on the results in human cells, isolates were classified in two groups: SXB_BS.0204 isolates, that exhibited reduced invasion, and CRP isolates, that exhibited normal invasion in human epithelial cells.

### *Salmonella* Derby SXB_BS.0204 PFGE profile forms a distinct phylogenetic clade

**Figure 2:**
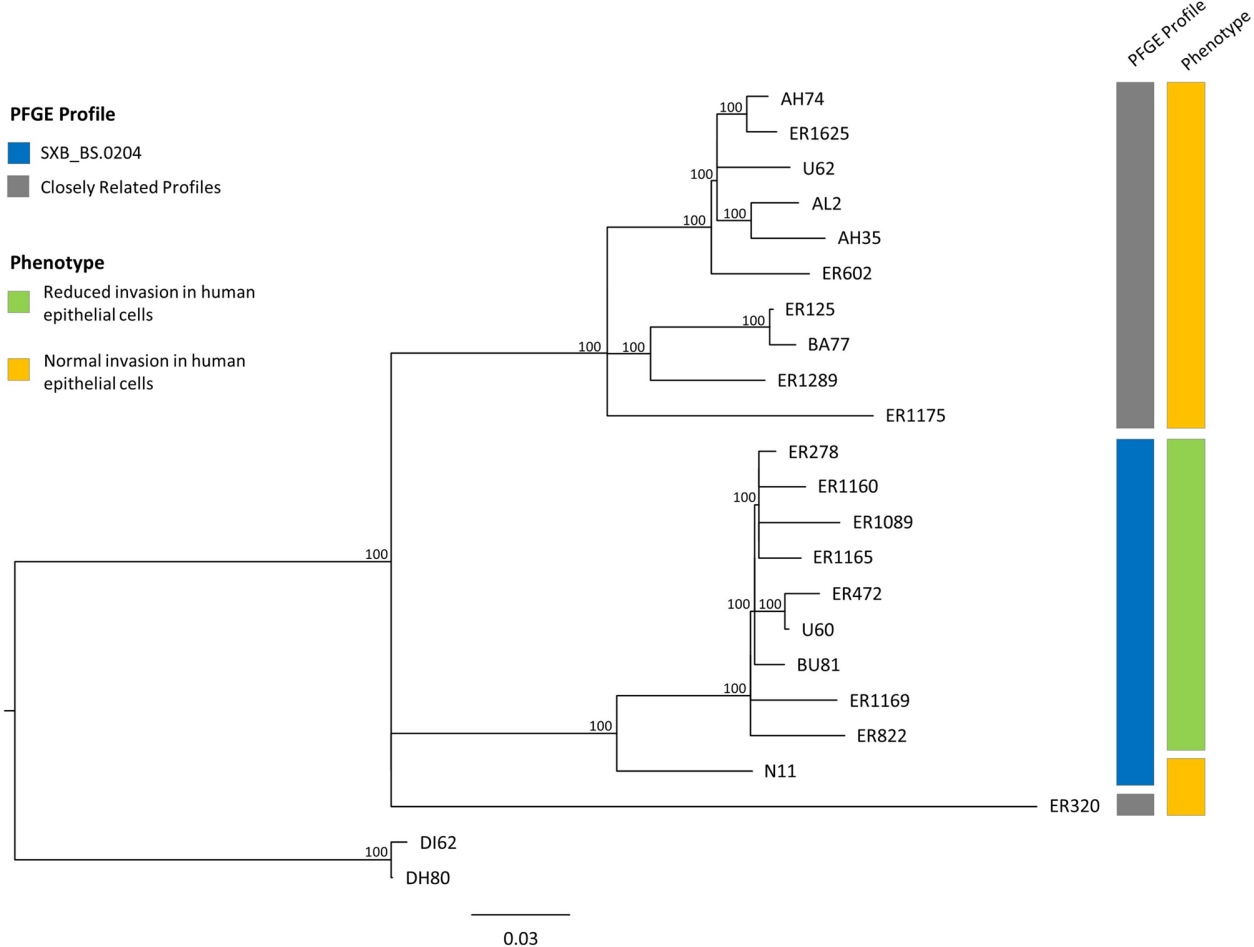
Maximum-likelihood phylogenetic tree of representative *S*. Derby isolates of the study. PFGE type and the virulence phenotype are shown in the filled boxes as indicated in the embedded key. Isolates DI62 and DH80 (SXB_PR.0072 profile) were selected as outgroup. Bootstrap values of the nodes are reported when > 70.

To evaluate the phylogenetic relationship of *S*. Derby isolates with distinct interaction with epithelial cells, the whole genome sequence of 10 SXB_BS.0204 isolates and 11 CRPs was determined. Two *S*. Derby isolates with PFGE profile SXB_PR.0072 that was distantly related to SXB_BS.0204 and CRPs, were also included as an outgroup to provide phylogenetic context (Supplementary Table 2). All isolates were sequence type 40 (ST40), typical of *S*. Derby from swine^6^. A maximum-likelihood tree was constructed based on 893 SNPs in the core genome. Two clades contained most of the isolates, the first contained all the SXB_BS.0204 isolates with a mean pairwise SNP distance of 26, and the second all the CRP isolates, except ER320, with a mean pairwise SNP distance of 67. The two clades were closely related, with a mean pairwise SNP distance of 196 for strain ER1175 to SXB_BS.0204 isolates (Fig. 2). Strain ER320 was present on a separate similarly diverged lineage that shared a common ancestor with the SXB_BS.0204 and CRP clades.

### Differences in gene content do not result in decreased invasion of SXB_BS.0204 isolates

Host adaptation is commonly associated with genome degradation and horizontal gene transfer^1^. We thus determined genes exclusively present in the genomes of CRP isolates that were absent in SXB_BS.0204 isolates with reduced invasion in epithelial cells. Genes shared with SXB_BS.0204 isolate N11 were omitted as it was phylogenetically distinct from other SXB_BS.0204 isolates and had an intermediate phenotype in host cells. ER320 was also omitted since it was phylogenetically distinct from the other CRP isolates (Fig. 2). The resulting group of 10 CRP isolates shared 69 CDSs absent in SXB_BS.0204 isolates (Supplementary Table 3), located on the chromosome within *Salmonella* genomic island 1 (SGI-1)^15^ and other six genomic regions (here identified as B1–B6). Genomic regions B1–B6, and SGI-1 were deleted from ER1175, a representative CRP clade isolate, but no decrease in intracellular loads at 2 h and 22 h p.i. in human or swine epithelial cells was observed (Supplementary Fig. 2). We concluded that the decrease in invasion observed in SXB_BS.0204 isolates was not due to the loss of genomic regions B1 to B6, or SGI-1.

### Non-sense mutations in SXB_BS.0204 isolates do not explain their reduced invasion in human epithelial cells

The ability of *Salmonella* to specifically colonize different hosts may depend on allelic variation of virulence genes^10^. To determine if specific allelic variants were responsible for the different interaction with host epithelial cells observed for CRP isolates vs SXB_BS.0204 isolates, we identified the SNPs that discriminate the two groups. SNPs shared with SXB_BS.0204 isolate N11 were omitted as it was phylogenetically distinct from other SXB_BS.0204 isolates and had an intermediate phenotype in host cells. ER320 was also omitted since it was phylogenetically distinct from the other CRP isolates (Fig. 2). A total of 30 SNPs discriminated the two groups (Supplementary Table 5). Two SNPs are located in intergenic regions, 10 are synonymous, and 18 are non-synonymous. Among nsSNPs we found 6 conservative missense mutations, 10 non-conservative missense mutations and 2 non-sense mutations. The 2 non-sense mutations detected in the SXB_BS.0204 isolates are located in *ydiV*, encoding a repressor of flagellar class II operons^16,17^, and *yhaK*, encoding an unknown protein^18^. Both non-sense mutations (at position 367 in the 714 bp-*ydiV* coding sequence, at position 354 in the 702 bp-*yhaK* coding sequence) are located in the middle of the respective CDS, possibly resulting in significant functional alterations. The non-mutated allelic variants of *ydiV* and *yhaK* were replaced in ER1175 with the variants bearing the non-sense mutations, to obtain the strains ER1175::*ydiV_a* and ER1175::*yhaK_a*, respectively. Recombinant ER1175 strains did not exhibit decreased intracellular loads at 2 h and 22 h p.i. in human and swine epithelial cells, compared to ER1175 (Supplementary Fig. 3), thus demonstrating that the two non-sense mutations do not explain the reduced invasion observed for SXB_BS.0204 isolates.

### A single non-synonymous mutation in *hilD* is responsible for the reduced invasion of human epithelial cells of SXB_BS.0204 isolates

**Figure 3:**
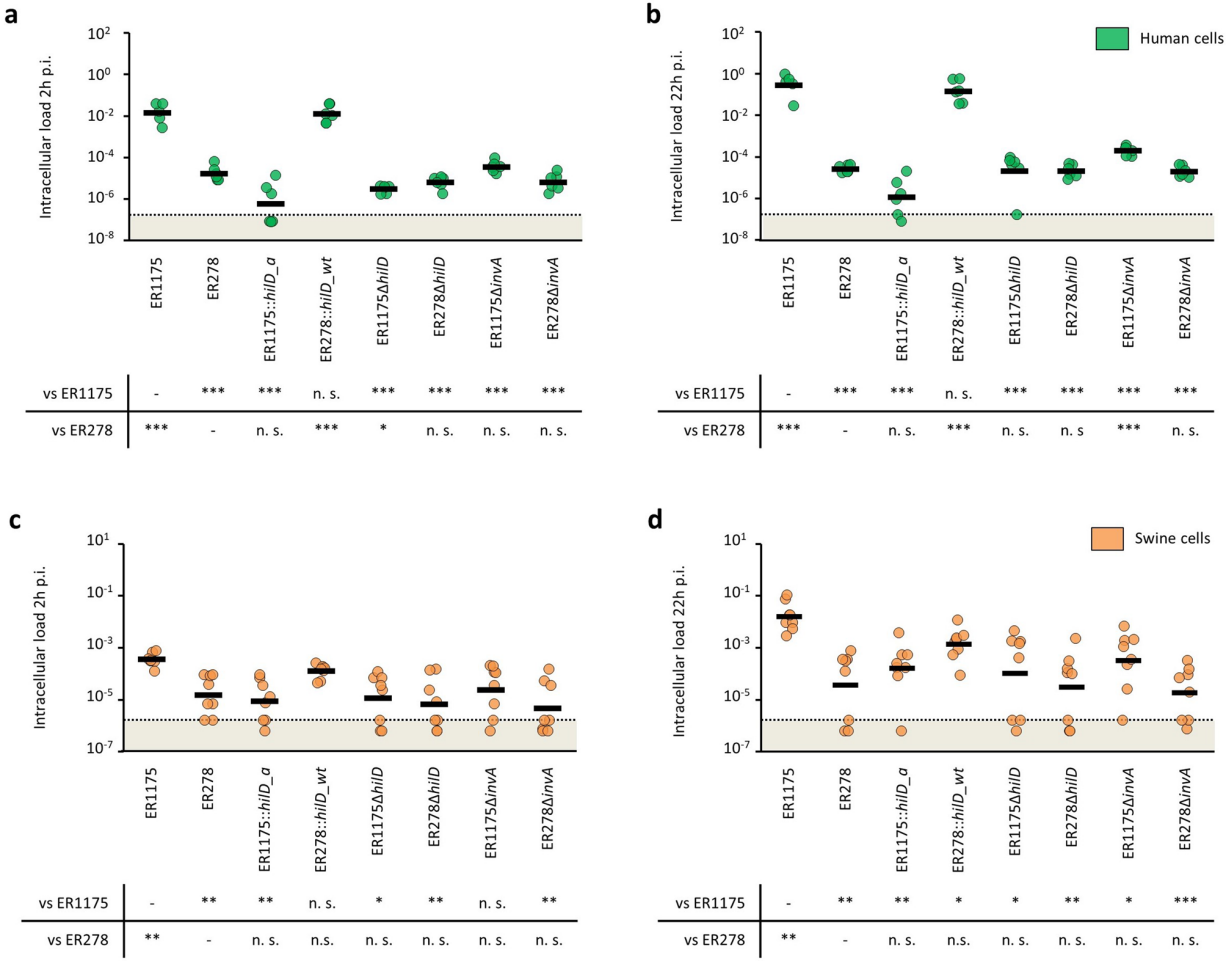
Role of *hilD* point mutation in virulence. Intracellular loads at 2 h and 22 h post infection (p.i.) of ER1175, ER278 and their mutants in human cells (**a**,**b**) and swine cells (**c**,**d**). Results are expressed as intracellular bacteria recovered after 2 h and 22 h of infection divided by the inoculum. Three biological replicates were performed in human cells, four replicates in swine cells and each tested in duplicate. Dots represent the values of the replicates. Arithmetic means are indicated by horizontal lines. Tables report p-values from two-tailed Student’s t-test corrected for multiple comparisons (*p < 0.05, **p < 0.01, ***p < 0.001, n.s. not significant). The dotted line represents the limit of detection.

One of the ten non-synonymous mutations was in the *hilD* gene that encodes the major activator of *Salmonella* pathogenicity island 1 (SPI-1). SPI-1 encodes a type three secretion system (T3SS-1), that translocates effector proteins into the host cell cytosol resulting in invasion^19,20^. The *hilD* allelic variant of the non-invasive isolates (*hilD_a*) has a TAT in position 291, coding for tyrosine, instead of a TGT coding for cysteine, present in CRP isolates (*hilD_wt*). This mutation was immediately downstream of an amino acid residue predicted to make base-specific contact with DNA^21^. To evaluate the SNP effect on *hilD* stability, we used the Structure-based stability change prediction upon single-point mutation method (STRUM)^22^ which predicts the protein fold stability change of HilD encoded by *hilD_a* as compared to *hilD_wt*. The protein fold stability change was measured calculating the free energy gap difference (∆∆G) between the mutant protein (HilD_a, Δ*G*_m_) and the wild type protein (HilD_wt, Δ*G*_w_), ΔΔG = Δ*G*_m_ − Δ*G*_w_^22^. The predicted ∆∆G was − 3.09, suggesting a strong destabilizing effect on the protein fold. To determine the effect of the polymorphism in *hilD*, we replaced *hilD_wt* of ER1175 with *hilD_a* (ER1175::*hilD_a*) and replaced *hilD_a* of ER278, a representative isolate of the SXB_BS.0204 with reduced invasion, with *hilD_wt* (ER278::*hilD_wt*). In human cells, (Fig. 3a,b) the intracellular loads at 2 h and 22 h p.i. of ER1175::*hilD_a* were respectively 4 and 5 logs lower than ER1175 (*p* < 0.001). Consistently, ER278::*hilD_wt* showed 3 to 4 logs higher intracellular loads at 2 h and 22 h p.i. than its wildtype strain (*p* < 0.001), with a phenotype comparable to ER1175. Conversely, in swine cells (Fig. 3c,d) the intracellular loads at 2 h and 22 h p.i. of ER1175::*hilD_a* were reduced by only 1–2 logs compared to ER1175 (*p* < 0.01) and ER278::*hilD_wt* was as invasive as its wild type strain. These results showed that the two *hilD* allelic variants are responsible for the different interaction with host human cells observed, but they do not fully explain the differences found in swine cell infection. To assess if the SXB_BS.0204 isolates were less invasive because *hilD_a* encodes a non-functional protein, we compared the intracellular loads at 2 h and 22 h p.i. of wild type ER278 with mutant ER1175 and ER278, both deleted for *hilD*. We found that ER1175Δ*hilD* and ER278Δ*hilD* invaded both human and swine cells to the same or slightly lower extent of ER278. These results suggest that SXB_BS.0204 isolates carry a loss-of-function mutation in *hilD*. Since HilD is the major activator of SPI-1, we evaluated if the loss of HilD functionality confers the same invasion phenotype of a complete loss of a functional T3SS-1. We thus deleted *invA,* that encodes a structural component of the T3SS-1, in ER1175 and ER278. ER1175Δ*invA* invaded both cell lines at the same level of ER278 and showed 1 log higher intracellular load at 22 h p.i. in human, but not in swine cells. ER278Δ*invA* had the same phenotype of ER278 in both cell lines. The results suggest that the point mutation in *hilD_a* leads to complete loss of functionality of T3SS-1. As T3SS-1 is also involved in *Salmonella* adhesion to host cell membrane^23^, we further analyzed the ability of the mutant strains to adhere to the two cell lines (Supplementary Fig. 4). Adhesion of ER1175 to human cells was significantly higher than that of ER1175::*hilD_a* and the mutants deleted for *hilD* and *invA*, but importantly ER278 adhered at a similar level to ER1175. In swine cells, no difference in adhesion was detected between ER1175, ER278 and the tested mutants. The loss of a functional *hilD* or a structural component of T3SS-1 in CRP isolates leads to impairment of adhesion to human cells whereas in SXB_BS.0204 isolates with reduced invasion this had no effect, suggesting that alternative mechanisms of adhesion are involved in each case. Together, these data show that losing the ability to express a functional HilD or T3SS-1 appears more detrimental to the entire infection process of human rather than swine cells.

### The *hilD_a* allele encodes a non-functional regulator

**Figure 4:**
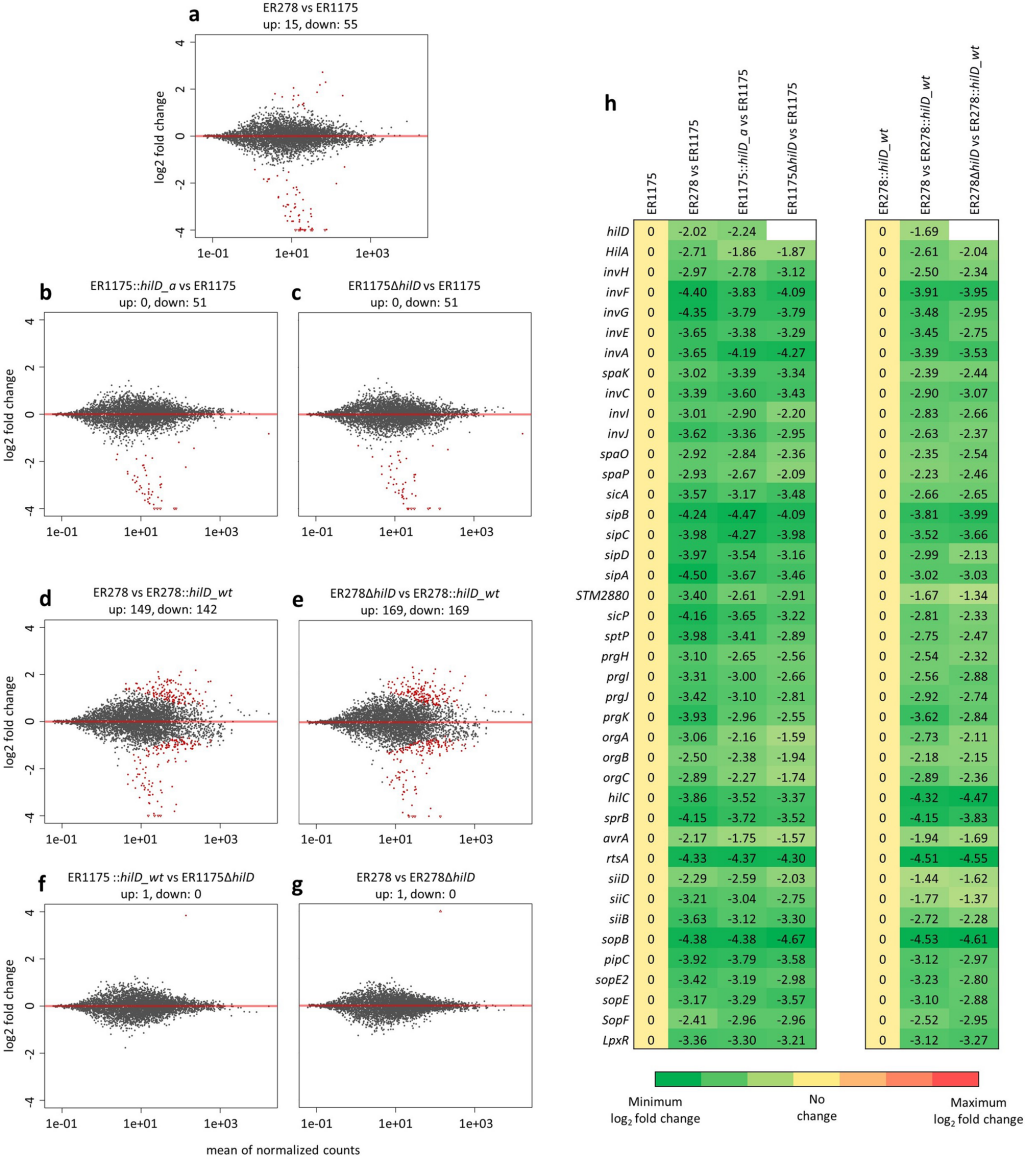
Comparative transcriptome analysis. (**a**–**g**) MA-plots. The gene expression log2 fold change for each comparison is plotted on the y-axis and the average of the counts normalized by size factor is shown on the x-axis. Each gene is represented by a dot. Genes with an adjusted p-value below the threshold (0.05) are shown in red. The number of up and down-regulated genes in each comparison is specified above the corresponding MA-plot. (**h**) List of genes significantly downregulated in strains carrying *hilD_a* or deleted for *hilD* compared to the same strains carrying *hilD_wt*. For each gene, name and log2 fold change are reported.

To test if *hilD_a* encoded a non-functional protein we compared the transcriptome of ER1175, ER278, ER1175::*hilD_a*, ER278::*hilD_wt*, ER1175Δ*hilD*, ER278Δ*hilD*. Total RNA was extracted from the strains grown to early stationary phase as it was previously reported that the high expression of the SPI-1 genes in this phase is reduced by an average of 40-fold in *S*. Typhimurium deleted for *hilD*^24^. The results of the differential expression analysis are summarized in seven pair-wise comparisons (Fig. 4a–g). We searched for genes downregulated in all strains carrying *hilD_a* or deleted for *hilD* (ER278, ER1175::*hilD_a*, ER1175Δ*hilD* and ER278Δ*hilD*) compared with all strains carrying *hilD_wt* (ER1175 and ER278::*hilD_wt*). We identified 30 SPI-1 genes and 10 genes encoded elsewhere but known either to be regulated by HilD or to have an expression profile comparable to those of SPI-1 genes (Fig. 4h), namely *rtsA*, involved in SPI-1 regulation^25^; 3 SPI-4 genes (*siiB*, *siiC, siiD*)^26^; *sopB* and *sigE*, located in SPI-5^27^, *sopF*^28^, *sopE* and *sopE2*^29^, encoding for T3SS-1 effectors; *lpxR*, encoding for an LPS-modifying enzyme^30^. The *hilD* gene was downregulated in strains carrying *hilD_a*, consistently with the fact that HilD activates the expression of its regulon as well as promoting its own transcription^31^. HilD is reported to also promote expression of flagellar genes by activating the flagellar operon *flhDC*^32^. However, we did not observe any significant downregulation of expression of *flhDC* and other flagellar genes in strains carrying *hilD_a* or deleted for *hilD* compared with the isogenic strains carrying *hilD_wt*. To examine the role of HilD in flagellar genes regulation in our strains, we evaluated the swarming and swimming motility of ER1175, ER278 and their *hilD* mutants (Supplementary Methods). No differences were observed in swimming motility (Supplementary Fig. S5a), whereas ER278 and its isogenic mutants showed an increased ability to swarm compared to ER1175 and its isogenic mutants (Supplementary Fig. S5b,c). These results indicate that swimming motility is uninfluenced by HilD in the studied strains and the differences in swarming ability are not due to the presence of a functional HilD. Therefore, *hilD* appears not to be crucial for flagellar genes expression in the studied strains.

No genes were differentially expressed in the pairs ER1175::*hilD_a*-ER1175Δ*hilD* and ER278-ER278Δ*hilD* (Fig. 4f,g). Overall the results confirm that the point mutation in *hilD_a* leads to a total loss of function of the encoded regulator.

### Lack of activation of SPI-1 expression during human cell infection caused by *hilD_a*

**Figure 5:**
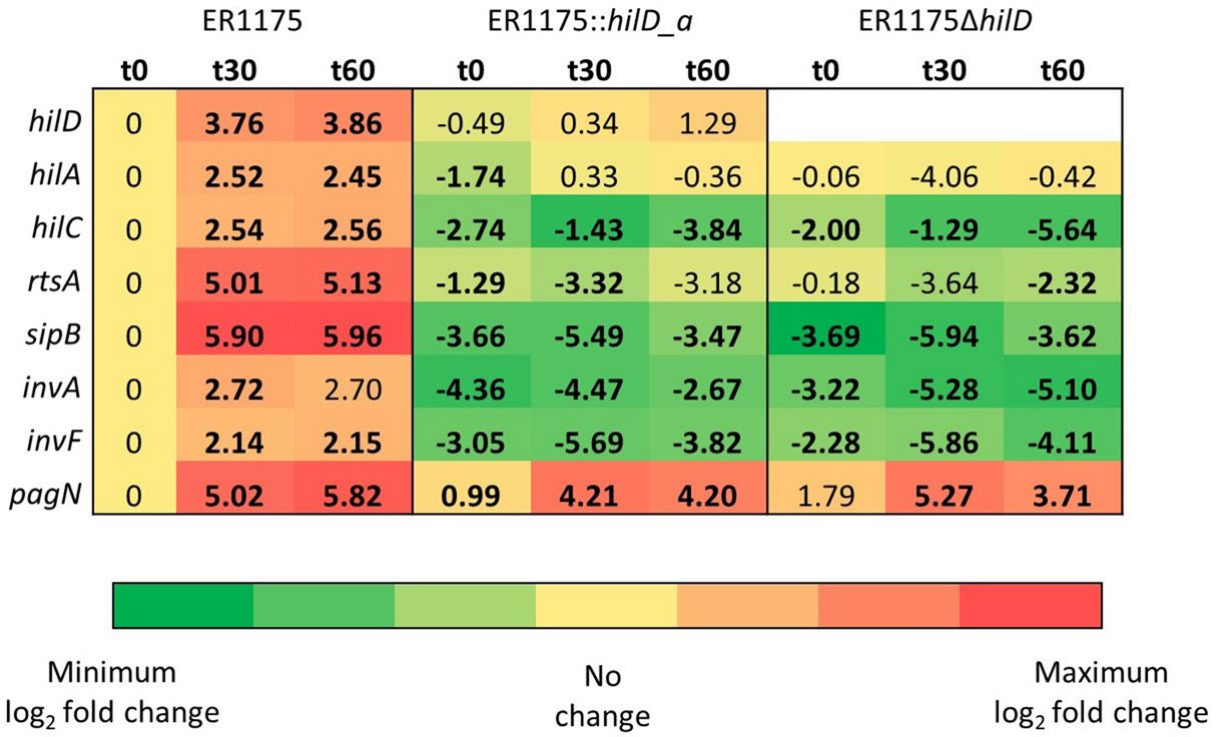
Gene expression during infection of human cells. Quantitative RT-PCR on ER1175 and its mutants during infection of human cells. The table reports the log2 fold-change values relative to ER1175-t0. Bold values are significantly different to ER1175-t0 (adjusted p-value < 0.05).

To evaluate if *hilD_a* resulted in the reduced expression of SPI-1 genes observed in broth culture conditions also during infection of human cells, bacterial RNA from ER1175, ER1175::*hilD_a* and ER1175Δ*hilD* was extracted at 0, 30 and 60 min post infection (t0, t30, t60) and qRT-PCR was performed for *hilD* and six other SPI-1 genes, namely the genes encoding the four transcriptional regulators HilC, RtsA, HilA, and InvF, the T3SS-1 structural component InvA, and the effector protein SipB (Supplementary Methods). In addition, *pagN* was included in the analysis as it mediates adhesion and invasion of epithelial cells without being directly regulated by HilD^33,34^ (Fig. 5). In ER1175 we observed an increased expression of all analyzed genes over time, up to 5.96 base-2 logs at t60. At t0, in both ER1175::*hilD_a* and ER1175Δ*hilD* the expression of SPI-1 genes was up to 4.36 base-2 logs lower than in ER1175, and no appreciable increase in gene expression was detected at t30 and t60. As expected, *pagN* was upregulated also in ER1175::*hilD_a* and ER1175Δ*hilD* at t30 and t60, but, interestingly, at t60 *pagN* expression was between 1.62 and 2.11 base-2 logs lower in the mutants compared to the wild type strain (*p* < 0.05), suggesting that HilD could be involved in the amplification of *pagN* expression. Taken together, these results show that *hilD_a* encodes for a non-functional regulator as observed in broth culture conditions and demonstrate that the absence of a functional HilD causes the lack of activation of SPI-1 expression over time during human cell infection.

### Intracellular replication phenotype differs between *S*. Derby strains carrying different *hilD* alleles

**Figure 6:**
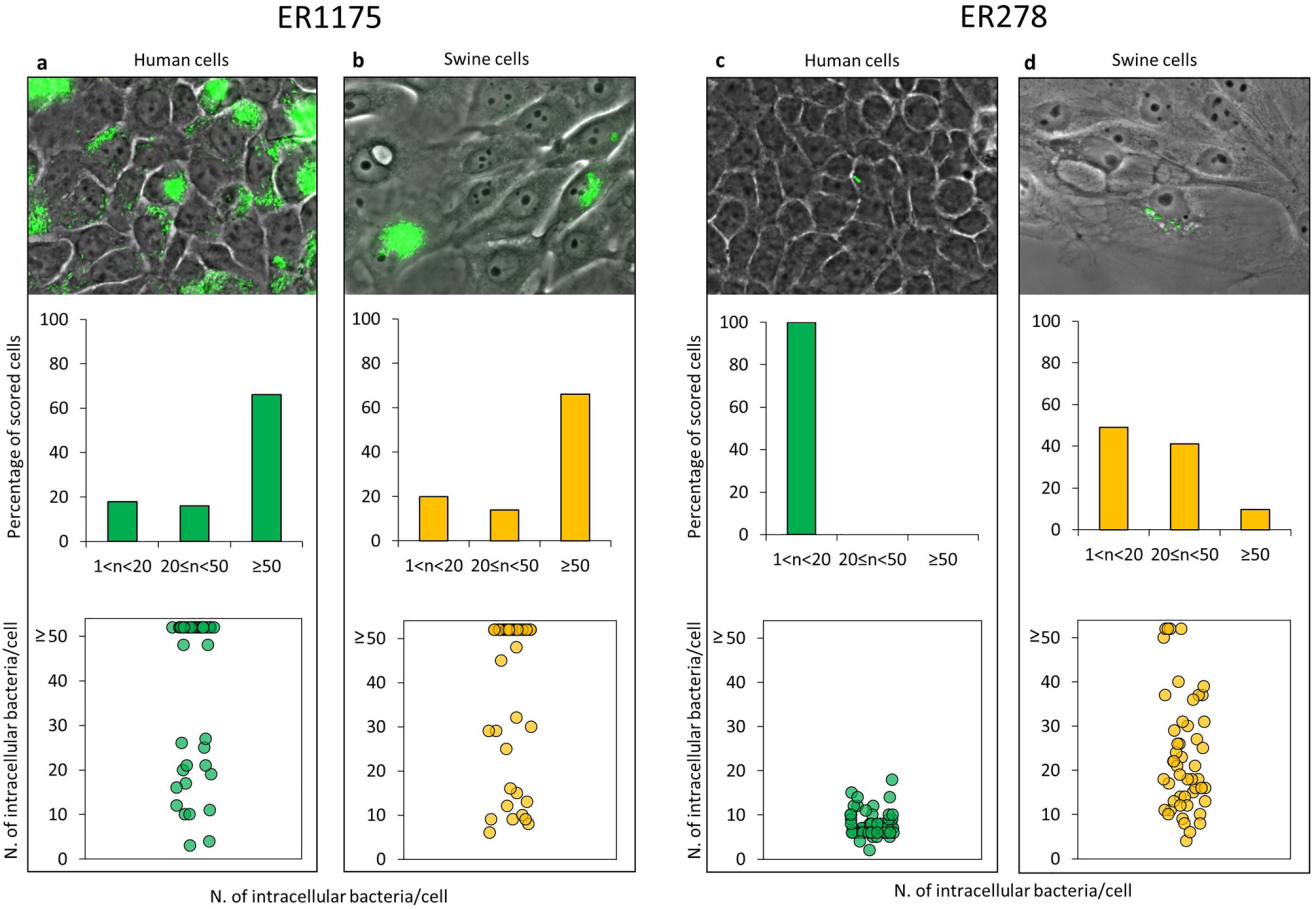
Intracellular replication phenotype of ER1175 and ER278 strains in human and swine cells. Each panel (**a**–**d**) contains: a representative image of intracellular replication of *S*. Derby representative strains ER1175 and ER278; a graph displaying the percentage of infected cells carrying 1–20, 20–50 and ≥ 50 Salmonellae 24 h post infection; a graph reporting the number of internalized bacteria per cell (each dot represents one infected cell). ≥ 50 infected cells were scored for each condition.

Two replicative phenotypes were reported for *Salmonella* upon invasion of epithelial host cells: (1) intravacuolar slow replication within *Salmonella*-containing vacuoles (SCVs) and (2) SPI-1 induced cytosolic hyper-replication^35^. Therefore, we used single-cell analysis by fluorescent microscopy to evaluate if ER1175 and ER278, carrying different *hilD* alleles, differ in their ability to replicate within human or swine cells. As it was reported that cells containing more than 50 bacteria can be indicative of the hyper-replicating phenotype^36^, we calculated the statistical differences in occurrence of cells containing ≥ 50 bacteria between the two strains. The majority of human and swine cells infected by ER1175 carried more than 50 bacteria (Fig. 6a,b), whereas no or few ER278-infected cells reached that bacterial load (Fig. 6c,d) (*p* < 10^–6^).

These results are in line with the previous observation that SPI-1 is important for intracellular hyper-replication^35^.

We observed that ER278 behaved differently in the two cell lines. ER278-infected human cells carried no more than 20 bacteria/cell, indicating that ER278, carrying a non-functional *hilD* allele, cannot hyper-replicate in human cells. Differently, several swine-infected cells contained 20–50 bacteria, and even cells with more than 50 bacteria were detected (Fig. 6d). This could be explained by differences between the two cell types or by the use of a mechanism alternative to SPI-1 by ER278 in swine cells.

## Discussion

The remarkable adaptation of *S*. Derby SXB_BS.0204 isolates to swine compared with humans, indicated by the epidemiological records, correlated with its significantly reduced invasion in human epithelial cells as opposed to swine cells. Phylogenetic analysis confirmed that SXB_BS.0204 isolates form a tight monophyletic group pointing to the existence of peculiar genetic determinants behind their further adapted phenotype. In SXB_BS.0204 we identified one non-conservative missense mutation in *hilD*, the major activator of SPI-1, and demonstrated that in human cells the *hilD_a* allele induces the same phenotype of a complete *hilD* deletion or the absence of a functional T3SS-1, indicating that *hilD_a* encodes for a nonfunctional protein. In swine cells *hilD_a* attenuated phenotype was only marginally observed indicating that HilD strongly conditions *Salmonella* invasion in human cells but only partly in swine cells. These results confirm the prevailing paradigm of an essential SPI-1 role for epithelial invasion in human cells but do not support the same essential role in swine cells. Therefore, the SPI-1 pathway, considered essential for the pathogenesis of *Salmonella* infection, does not explain the infection of swine cells, suggesting that an alternative mechanism is involved in this host. Naberhaus et al.^37^ evaluated some aspects of the pathogenesis of Typhimurium, Derby and 4,[5],12:i: *Salmonella* strains through experimental infection of piglets. They detected *S*. Derby in the faeces of infected animals up to 21 days post infection, suggesting that *S*. Derby settles in the porcine intestine after infection. In addition, they observed that at 2 and 4 days post infection almost all infected piglets had detectable levels of *Salmonella* in their colon upon necropsy and tissue culture, but gross lesions suggestive of inflammation were absent in *S*. Derby-infected pigs as opposed to the pigs infected with *S*. Typhimurium and *S*. 4,[5],12:i:. These data could be indicative that *S*. Derby does not invade the intestinal epithelium to a sufficient extent to cause an observable inflammation, which is usually the result of the induction of several SPI-1 effectors. From this observation alone it could be argued that *S*. Derby does not need an active invasion of the epithelium to survive and replicate within the swine intestine, but Naberhaus et al. also showed that *S*. Derby reached the lymphatic system at a similar level as *S*. Typhimurium and *S*. 4,[5],12:i:. This observation implies the ability of the bacterium to cross the intestinal barrier in order to reach the lymph and hence reinforces our hypothesis that *S*. Derby indeed interacts and invades the intestinal epithelium, probably through an alternative invasion mechanism different from the classical SPI-1 induced inflammation. A similar conclusion was reported recently by Roche et al.^38^ who showed that a *S.* Typhimurium strain invalidated for known invasion factors has different virulence phenotypes in cell lines of different animal species. Remarkably, our results show that changes in host adaptation at the population level (swine vs human) may occur due to a single nsSNP in the master regulator of SPI-1. This finding is consistent with other studies that reported mechanisms of host-adaptation associated with nsSNPs, in particular in relation to changes in individual surface or effector proteins involved in recognition and invasion of host cells^10,39^. Similarly, a SNP in a noncoding promoter region was recently deemed responsible for high expression levels of the PgtE outer membrane protease and thus for the increased virulence of the epidemic African *S.* Typhimurium ST313^40^. The CRP isolate ER1175, carrying *hilD_wt,* showed a hyper-replicative behavior in both human and swine cells, whereas the SXB_BS0204 isolate ER278, carrying *hilD_a* maintained only a moderate ability to replicate inside swine cells while almost no replication was observed in human cells. These findings are consistent with the previous report that the T3SS-1 have a post-invasion role in the hyper-replication of *Salmonella* at least in human cells. At the same time, according to our results the same role appears less critical in swine cells. In conclusion, our results demonstrate that a single nsSNP in the major SPI-1 regulator gene *hilD* can play an important role in shaping the host adaptation profile of the *Salmonella* Derby population considered in our study where the mutated *hilD_a* allele is associated with the second most abundant PFGE type in swine, which in turn is almost absent in humans. Our findings could indicate that the loss of expression of SPI-1 due to the mutated *hilD_a* allele represents a further step in the adaptation of *S*. Derby to swine, leading to the fixation of the observed mutation.

Improving our knowledge on how *Salmonella* selectively invades and persists in different hosts will provide the foundation for more specific attribution of human infections to the different animal reservoirs. Moreover, a better understanding of the molecular pathogenesis and ecology of *Salmonella* across its different hosts will help adopt more refined mitigation measures for this pathogen at the population level.

## Methods

### Typing and distribution assessment of Salmonella isolates

*Salmonella* isolates were serotyped according to the Kauffmann–White-Le Minor (KW) scheme^41^ and genotyped by Pulsed-Field Gel Electrophoresis following the PulseNet Standard Operating Procedure^42^. PFGE patterns were analysed using the BioNumerics Software v6.6 (Applied Maths NV, bioMérieux). Clustering of PFGE patterns was done using the Unweighted Pair Group Method with Arithmetic Average (UPGMA)^14^ based on the Dice similarity index (optimization, 1%; band matching tolerance, 1%) for PFGE type assignment. The selection ratio (SR) was calculated to quantify the distribution disequilibrium of *S*. Derby PFGE types between the human and swine populations. The SR is defined as the proportional use of a resource divided by its proportional availability. In this study, the SR correspond to the ratio between the number of human isolates of a specific PFGE type over all human isolates of *S*. Derby and the number of swine isolates of the same type over all swine isolates of *S*. Derby. Multinomial simulations were performed to evaluate if SRs of different PFGE types were significantly different from 1.

### Cell culture infection assays

Human INT-407 (source: American Type Culture Collection—CCL-6) and swine IPEC-J2 (source: Institut fur Mikrobioòogie und Tierseuchen, Freie, Universitat Berlin) intestinal epithelial cell lines were used for the in vitro cell culture infection assays. INT-407 cells were propagated in DMEM containing 10% fetal bovine serum, penicillin 100 U/mL and streptomycin 100 µg/mL. IPEC-J2 cells were cultured in 50% Dulbecco’s Modified Eagle’s Medium (DMEM) and 50% Ham’s F12 Nutrient Mixture containing 5% fetal bovine serum, penicillin 100 U/mL and streptomycin 100 µg/mL. Both cell lines were maintained at 37 °C in 5% CO_2_. For cell culture infection assays, IPEC-J2 and INT-407 cell lines were seeded in 96-well plates and incubated for 48 h to reach 100% confluency, then monolayers were washed twice and incubated with antibiotics-free medium for 1 h before bacterial inoculation. Bacterial isolates were grown statically to stationary phase at 37 °C in LB broth for inoculation. Each cell line was infected at a multiplicity of infection (MOI) of 10. *S.* Typhimurium LT2 and *S.* Typhimurium SL1344 strains were used as positive controls of infection, whereas *E. coli* BW25113 strain was used as negative control of infection. After inoculation, cell cultures were incubated at 37 °C with 5% CO_2_ for 1 h. For the adhesion assay, cell monolayers were then washed five times with fresh medium and disrupted with 0.1% Tryton X-100 for 30 min to score the number of adhered *Salmonellae*. For the determination of intracellular load 2 h post infection, cell cultures were incubated for 1 h of infection and then washed twice and incubated for another 1 h with fresh medium containing 100 μg/mL gentamycin to kill extracellular bacteria. Cell monolayers were then washed twice with PBS and lysed as for the adhesion assay. For the determination of intracellular load 22 h post infection, cell growth medium containing 100 μg/mL gentamycin was replaced after 1 h with fresh medium containing 10 μg/mL gentamycin to maintain clearance of extracellular *Salmonella* in the medium and cell cultures were further incubated for 20 h after which monolayers were lysed as for the adhesion assays. Serial tenfold dilutions of disrupted cell suspensions were plated on LB agar and incubated at 37 °C overnight for bacterial cell count. The adhesion rate was calculated as the number of bacteria recovered after 1 h of infection divided by the number of bacteria added as inoculum. The intracellular load 2 h post infection was calculated as the number of bacteria recovered after 1 h of infection upon gentamycin treatment divided by the number of bacteria added as inoculum. The intracellular load 22 h post infection was calculated as the number of bacteria recovered after 22 h of infection divided by the number of bacteria added as inoculum. Each experiment was repeated on different days (biological replicates) and performed in duplicate (technical replicates). The initial infection assays on 20 isolates belonging to SXB_BS.0204 and 18 isolates belonging to closely related PFGE types were performed in two biological replicates, whereas the assays done on the mutants of *hilD* were performed in three biological replicates. Statistical analysis was made on log-transformed data to guarantee errors normality. Data were analyzed with two-tailed Student’s *t*-test. The Bonferroni correction was applied for multiple comparisons.

### Whole genome sequencing and bioinformatics

Twenty-three isolates of *S*. Derby were whole-genome-sequenced (Supplementary Table 2). The isolates were cultured overnight at 37 °C under agitation (200 rpm) in Brain Heart Infusion (BHI) broth. Genomic DNA was extracted by Qiagen DNeasy blood and tissue kit, following the kit instructions but extending the incubation with Proteinase K to 20 h to enhance cells lysis. Sequencing libraries were prepared with the Nextera XT V2 sample preparation kit and sequenced using the MiSeq Reagent Kit v2 on an Illumina MiSeq platform, with a 2 × 250-bp paired-end run. Each reads set was evaluated for sequence quality and read-pair length using FastQC v0.11.9^43^ (https://qubeshub.org/resources/fastqc). The sequence type (ST) of each isolate was determined using the MLST Analysis Tool available online in EnteroBase v1.1.2 (https://enterobase.warwick.ac.uk/species/senterica/allele_st_search). Single Nucleotide Polymorphisms (SNPs) of each isolate were identified using SNIPPY v4.4.5^44^ (https://github.com/tseemann/snippy) with U60 isolate genome used as reference. Reads of the reference genome were de novo assembled by SPAdes v3.12.0^45^ (https://cab.spbu.ru/software/spades/), checked with QAST v4.6^46^ (http://quast.sourceforge.net/) and contigs (> 1000 bp) were re-ordered on *S*. Typhimurium LT2 genome by MAUVE v2.4.0^47^ (http://darlinglab.org/mauve/mauve.html). Gubbins v2.3.4^48^ (http://sanger-pathogens.github.io/gubbins/) was used to exclude SNPs in suspected recombined sequences from the analysis. The obtained core SNPs alignment was used to generate a Maximum-Likelihood phylogenetic tree by RAxML v7.0.4^49^ (https://cme.h-its.org/exelixis/web/software/raxml/), using a General Time-Reversible (GTR) substitution model with gamma correction for among-site rate variation. Support for nodes on the trees was assessed through 100 bootstrap replicates. For the orthology analysis, reads of virulent and attenuated isolates were de novo assembled using SPAdes v3.12.0^45^ (https://cab.spbu.ru/software/spades/) and protein-coding genes were predicted by Prodigal v2.6.0^50^ (https://anaconda.org/bioconda/prodigal). OrthoMCL v1.4^51^ (https://orthomcl.org/orthomcl/?rm=orthomcl) was used to find classes of orthologous genes. The output matrix was analyzed to select exclusive genes of virulent isolates. The selected genes were identified by mapping their sequences to the reference genome (N11 or ER1175, used as reference for virulent isolates) annotated by RAST v2.0^52^ (https://rast.nmpdr.org/). PlasmidFinder v2.0^53^ (https://cge.cbs.dtu.dk/services/PlasmidFinder-2.0/) was used for plasmid identification. To predict the protein fold stability change (ΔΔG) of the mutated HilD of attenuated isolates, we used the software STRUM^22^ (https://zhanglab.ccmb.med.umich.edu/STRUM/).

### Construction of recombinant strains

All primers used are described in Supplementary Table 6 and mutants generated in Supplementary Table 4. Gene deletion and allelic exchange were made using the lambda Red recombinase system, based on methods described previously by Datsenko and Wanner^54^. Details are provided in Supplementary Methods.

### Transcriptome investigation: RNA extraction and sequencing

For transcriptome analysis, *Salmonella* strains were grown overnight in 5 mL LB broth and diluted to OD_600_ = 0.1 in 20 mL LB broth in 250 mL flasks. Flasks were incubated at 37 °C and 220 rpm until OD_600_ = 2.0, then a 0.6 mL aliquot of each culture (about 1.2 × 10^9^ CFU/mL) was centrifuged at 16,000*g* for 2 min and pellets were frozen in a dry ice ethanol bath. Cells were resuspended in the Lysis Buffer of the FastRNA SPIN Kit for Microbes and processed in a FastPrep-24 Instrument for 90 s, at 6.0 m/s, for cell disruption. Trace DNA was removed by DNase digestion with TURBO DNA-free kit, using “rigorous” treatment. Complete loss of genomic DNA after DNase treatment was verified by PCR on RNA-samples using the following primers: rpoD_FW and rpoD_RV. RNA samples were then precipitated for purification. RNA quality was assessed using a 2100 Bioanalyzer according to Agilent RNA 6000 Nano Kit protocol. Each sample was then retro-transcribed using a SuperScript VILO cDNA Synthesis Kit. In order to obtain double-strand cDNA for library preparation, single-strand cDNA was purified on 1.8× Agencourt AMPure XP Beads and the second cDNA strand was synthesized using NEBNext Ultra II Non-Directional RNA Second Strand Synthesis. A second step of beads-based purification was performed before library preparation. RNA-Seq libraries were prepared starting from cDNA using Nextera XT V2. NextSeq 500/550 Mid Output v2 kit (2 × 150 cycles) was used for the sequencing run. Three independent biological replicates of RNA from each strain were sequenced.

### Transcriptome investigation: gene expression analysis

The genomic reads of isolate ER1175 were assembled by SPAdes v3.12.0^45^ (https://cab.spbu.ru/software/spades/) and checked with QAST v4.6^46^ (http://quast.sourceforge.net/) to generate the reference genome for transcriptome analysis. Contigs were concatenated and annotated by RAST v2.0^52^ (https://rast.nmpdr.org/). The genomic reads of isolate ER278 were mapped to ER1175 concatenated contigs in order to find and remove genes absent in ER278. ER1175 genes covered by less than 99% by ER278 reads were removed from differential expression analysis. Quality checked transcriptomic reads from each sample were mapped to ER1175 draft genome by HISAT v2.0.4^55^ (http://daehwankimlab.github.io/hisat2/). HTseq v0.9.1^56^ (https://htseq.readthedocs.io/en/release_0.9.1/) was used to estimate the number of reads of each sample mapped on the annotated genes. Detection of differentially expressed genes was made using the package DESeq2 v1.28.1^57^ (https://bioconductor.org/packages/release/bioc/html/DESeq2.html).

### Fluorescence imaging

Epithelial cells were seeded 48 h prior to infection on coverslips placed into 6-well tissue culture plates, at a density of 1 × 10^5^ cells/well for IPECJ2 cells and of 3 × 10^5^ cells/well for INT407 cells. *S*. Derby strains carrying mEGFP-pBAD plasmid (gift from Michael Davidson-Addgene plasmid # 54622) were grown to stationary phase in LB broth with Ampicillin (100 µg/mL). The cell culture infection assay protocol was followed with some modifications. Expression of mEGFP was induced both in broth 1 h prior to infection with l-arabinose (1.5 mg/mL) and inside mammalian cells maintaining l-arabinose (10 mg/mL) in the culture medium throughout infection. Monolayers were infected at MOI 50 and washed with PBS 24 h post infection, then fixed with paraformaldehyde (PFA) 4% for 20 min at room temperature. INT-407 and IPECJ2 cells were labeled respectively with Evans Blue 0.005% and HCS CellMask™ Blue stain, to enhance resolution in phase-contrast imaging. Samples were imaged on a Carl Zeiss Axio Scope A1 polarized light microscope, equipped with AxioCam ICm1. ZEN2012 Blue Edition (https://www.zeiss.com/microscopy/int/products/microscope-software/zen.html) software was used for acquisition and figures were assembled with FIJI v1.52i^58^ (https://imagej.net/Fiji). Statistical differences in the occurrence of host cells containing ≥ 50 Salmonellae were evaluated through Fisher's exact tests.

## Supplementary information

Supplementary Information 1.

Supplementary Information 2.

Supplementary Information 3.

Supplementary Information 4.

Supplementary Information 5.

## Data Availability

All genomic sequences produced in this study are publicly available on EBI under Project number PRJEB34865 and sample accession numbers are available in Supplementary Table 1. The ER1175 assembled and annotated genome is available under sample accession number ERS3898662. All RNA-seq data has been deposited in NCBI’s Gene Expression Omnibus under accession number GSE142220.

## References

[1] Kingsley RA, Bäumler AJ (2000). Host adaptation and the emergence of infectious disease: The *Salmonella* paradigm. Mol. Microbiol..

[2] Bäumler AJ, Fang FC (2013). Host specificity of bacterial pathogens. Cold Spring Harb. Perspect. Med..

[3] Branchu P, Bawn M, Kingsley RA (2018). Genome variation and molecular epidemiology of *Salmonella enterica* serovar Typhimurium Pathovariants. Infect. Immun..

[4] Kingsley RA (2013). Genome and transcriptome adaptation accompanying emergence of the definitive type 2 host-restricted *Salmonella enterica* serovar Typhimurium pathovar. Mbio.

[5] Mather AE (2016). Genomic analysis of *Salmonella enterica* serovar Typhimurium from wild passerines in England and Wales. Appl. Environ. Microbiol..

[6] Sévellec Y (2018). Polyphyletic nature of *Salmonella enterica* serotype Derby and lineage-specific host-association revealed by genome-wide analysis. Front. Microbiol..

[7] EFSA. The European Union One Health 2018 Zoonoses Report (2019). 10.2903/j.efsa.2019.5926 PMC7055727 32626211

[8] CDC. *Salmonella* Serotypes Isolated from Animals and Related Sources (2016).

[9] CDC. National enteric disease surveillance: *Salmonella* Annual Report, 2016 (2018).

[10] Yue M (2015). Allelic variation contributes to bacterial host specificity. Nat. Commun..

[11] Holt KE (2009). Pseudogene accumulation in the evolutionary histories of *Salmonella enterica* serovars Paratyphi A and Typhi. BMC Genom..

[12] Hayward MR (2014). SPI-23 of *S.* Derby: Role in adherence and invasion of porcine tissues. PLoS ONE.

[13] Ellermeier CD, Ellermeier JR, Slauch JM (2005). HilD, HilC and RtsA constitute a feed forward loop that controls expression of the SPI1 type three secretion system regulator hilA in *Salmonella* enterica serovar Typhimurium. Mol. Microbiol..

[14] Sneath PHA, Sokal RR (1973). Numerical Taxonomy. The Principles and Practice of Numerical Classification.

[15] Doublet B, Boyd D, Mulvey MR, Cloeckaert A (2005). The *Salmonella* genomic island 1 is an integrative mobilizable element. Mol. Microbiol..

[16] Wada T (2011). EAL domain protein YdiV acts as an anti-FlhD4C2 factor responsible for nutritional control of the flagellar regulon in *Salmonella enterica* Serovar Typhimurium. J. Bacteriol..

[17] Hisert KB (2005). A glutamate-alanine-leucine (EAL) domain protein of *Salmonella* controls bacterial survival in mice, antioxidant defence and killing of macrophages: Role of cyclic diGMP. Mol. Microbiol..

[18] Gurmu D (2009). The crystal structure of the protein YhaK from *Escherichia coli* reveals a new subclass of redox sensitive enterobacterial bicupins. Proteins.

[19] Galán JE, Curtiss R (1989). Cloning and molecular characterization of genes whose products allow *Salmonella* Typhimurium to penetrate tissue culture cells. Proc. Natl. Acad. Sci. U. S. A..

[20] Zhang K (2018). Minimal SPI1-T3SS effector requirement for *Salmonella* enterocyte invasion and intracellular proliferation in vivo. PLoS Pathog..

[21] Petrone BL, Stringer AM, Wade JT (2013). Identification of HilD-regulated genes in *Salmonella enterica* Serovar Typhimurium. J. Bacteriol..

[22] Quan L, Lv Q, Zhang Y (2016). STRUM: Structure-based stability change prediction upon single-point mutation. Bioinformatics.

[23] Lara-Tejero M, Galan JE (2009). *Salmonella enterica* serovar Typhimurium pathogenicity island 1-encoded type III secretion system translocases mediate intimate attachment to nonphagocytic cells. Infect. Immun..

[24] Colgan AM (2016). The impact of 18 ancestral and horizontally-acquired regulatory proteins upon the transcriptome and sRNA landscape of *Salmonella enterica* serovar Typhimurium. PLoS Genet..

[25] Ellermeier CD, Slauch JM (2003). RtsA and RtsB coordinately regulate expression of the invasion and flagellar genes in *Salmonella enterica* serovar Typhimurium. J. Bacteriol..

[26] Main-Hester KL, Colpitts KM, Thomas GA, Fang FC, Libby SJ (2008). Coordinate regulation of *Salmonella* pathogenicity island 1 (SPI1) and SPI4 in *Salmonella enterica* serovar Typhimurium. Infect. Immun..

[27] Darwin KH, Miller VL (2000). The putative invasion protein chaperone SicA acts together with InvF to activate the expression of *Salmonella* Typhimurium virulence genes. Mol. Microbiol..

[28] Cheng S (2017). Identification of a novel *Salmonella* type III effector by quantitative secretome profiling. Mol. Cell. Proteom..

[29] Bakshi CS (2000). Identification of SopE2, a *Salmonella* secreted protein which is highly homologous to SopE and involved in bacterial invasion of epithelial cells. J. Bacteriol..

[30] Kroger C (2013). An infection-relevant transcriptomic compendium for *Salmonella enterica* Serovar Typhimurium. Cell Host Microbe.

[31] Saini S, Ellermeier JR, Slauch JM, Rao CV (2010). The role of coupled positive feedback in the expression of the SPI1 type three secretion system in *Salmonella*. PLoS Pathog..

[32] Singer HM, Kuhne C, Deditius JA, Hughes KT, Erhardt M (2014). The Salmonella Spi1 virulence regulatory protein HilD directly activates transcription of the flagellar master operon flhDC. J. Bacteriol..

[33] Lambert MA, Smith SG (2008). The PagN protein of *Salmonella enterica* serovar Typhimurium is an adhesin and invasin. BMC Microbiol..

[34] Lambert MA, Smith SG (2009). The PagN protein mediates invasion via interaction with proteoglycan. FEMS Microbiol. Lett..

[35] Knodler LA (2010). Dissemination of invasive *Salmonella* via bacterial-induced extrusion of mucosal epithelia. Proc. Natl. Acad. Sci. U. S. A..

[36] Chong A, Starr T, Finn CE, Steele-Mortimer O (2019). A role for the *Salmonella* type III secretion system 1 in bacterial adaptation to the cytosol of epithelial cells. Mol. Microbiol..

[37] Naberhaus SA (2020). Pathogenicity and competitive fitness of *Salmonella enterica* serovar 4,[5],12:i: compared to *Salmonella* Typhimurium and *Salmonella* Derby in swine. Front. Vet. Sci..

[38] Roche SM (2018). *Salmonella* Typhimurium invalidated for the three currently known invasion factors keeps its ability to invade several cell models. Front. Cell. Infect. Microbiol..

[39] Eswarappa SM (2008). Differentially evolved genes of *Salmonella* Pathogenicity Islands: Insights into the mechanism of host specificity in *Salmonella*. PLoS ONE.

[40] Hammarlöf DL (2018). Role of a single noncoding nucleotide in the evolution of an epidemic African clade of *Salmonella*. Proc. Natl. Acad. Sci. U. S. A..

[41] Grimont PAD, Weill FX (2007). Antigenic Formulae of the *Salmonella* Serovars, 9th Revision.

[42] Standard Operating Procedure for PulseNet PFGE of *Escherichia coli* O157:H7, *Escherichia coli* non-O157 (STEC), *Salmonella* serotypes, *Shigella sonnei* and *Shigella flexneri* (2017).

[43] Andrews, S. FastQC: A quality control tool for high throughput sequence data. (2010) (https://qubeshub.org/resources/fastqc).

[44] Seemann, T. Snippy: Fast bacterial variant calling from NGS reads. (2015) (https://github.com/tseemann/snippy).

[45] Nurk S (2013). Assembling single-cell genomes and mini-metagenomes from chimeric MDA products. J. Comput. Biol..

[46] Gurevich A, Saveliev V, Vyahhi N, Glenn TG (2013). QUAST: Quality assessment tool for genome assemblies. Bioinformatics.

[47] Darling AC, Mau B, Blattner FR, Perna NT (2014). Mauve: Multiple alignment of conserved genomic sequence with rearrangements. Genome Res..

[48] Croucher NJ (2014). Rapid phylogenetic analysis of large samples of recombinant bacterial whole genome sequences using Gubbins. Nucleic Acids Res..

[49] Stamatakis A (2014). RAxML Version 8: A tool for phylogenetic analysis and post-analysis of large phylogenies. Bioinformatics.

[50] Hyatt D (2010). Prodigal: Prokaryotic gene recognition and translation initiation site identification. BMC Bioinform..

[51] Li L, Stoeckert CJ, Roos DS (2003). OrthoMCL: Identification of ortholog groups for eukaryotic genomes. Genome Res..

[52] Aziz RK (2008). The RAST server: Rapid annotations using subsystems technology. BMC Genom..

[53] Clausen PTLC, Aarestrup FM, Lund O (2018). Rapid and precise alignment of raw reads against redundant databases with KMA. BMC Bioinform..

[54] Datsenko KA, Wanner BL (2000). One-step inactivation of chromosomal genes in *Escherichia coli* K-12 using PCR products. Proc. Natl. Acad. Sci. U. S. A..

[55] Kim D, Langmead B, Salzberg SL (2015). HISAT: A fast spliced aligner with low memory requirements. Nat. Methods.

[56] Anders S, Pyl PT, Huber W (2014). HTSeq—A Python framework to work with high-throughput sequencing data. Bioinformatics.

[57] Love MI, Huber W, Anders S (2014). Moderated estimation of fold change and dispersion for RNA-seq data with DESeq2. Genome Biol..

[58] Schindelin J (2012). Fiji: An open-source platform for biological-image analysis. Nat. Methods.

