# Supplementary material for: Mutation of hilD in a Salmonella Derby lineage linked to swine adaptation and reduced risk to human health
Source: Sci Rep. 2020 Dec 9;10:21539. doi: 10.1038/s41598-020-78443-7 (PMC7726570; doi:10.1038/s41598-020-78443-7)
Supplement: Supplementary file 1 — Supplementary Information 1. [file 41598_2020_78443_MOESM1_ESM.pdf]

SUPPLEMENTARY MATERIALS

Mutation of hilD in a Salmonella Derby lineage linked to swine adaptation and reduced risk to human health

Martina Tambassi[1], Melissa Berni[1], Chiara Bracchi[1], Erika Scaltriti[1], Marina Morganti[1], Luca Bolzoni[1], Jennifer R. Tanner[2+], Gaëtan Thilliez[2], Robert A. Kingsley[2,3], Stefano Pongolini[1] & Gabriele Casadei[1,$,*]

[1]Risk Analysis and Genomic Epidemiology Unit, Istituto Zooprofilattico Sperimentale della Lombardia e dell'Emilia Romagna, Parma, Italy

[2]Quadram Institute Bioscience, Norwich Research Park, Colney, Norwich, UK

[3]University of East Anglia, Norwich, UK

[+]Present address: National Microbioloby Laboratory, Public Health Agency of Canada, Winnipeg, MB, Canada

[$] Present address: Regional Epidemiology Unit, Istituto Zooprofilattico Sperimentale della Lombardia e dell'Emilia Romagna, Bologna, Italy

*Correspondence: gabriele.casadei@izsler.it

This file contains:

Supplementary Figures S1 to S5

Supplementary Tables S1 and S4

Supplementary Tables S2, S3, S5, S6 are provided as separate tabular files

Supplementary Methods

Supplementary References

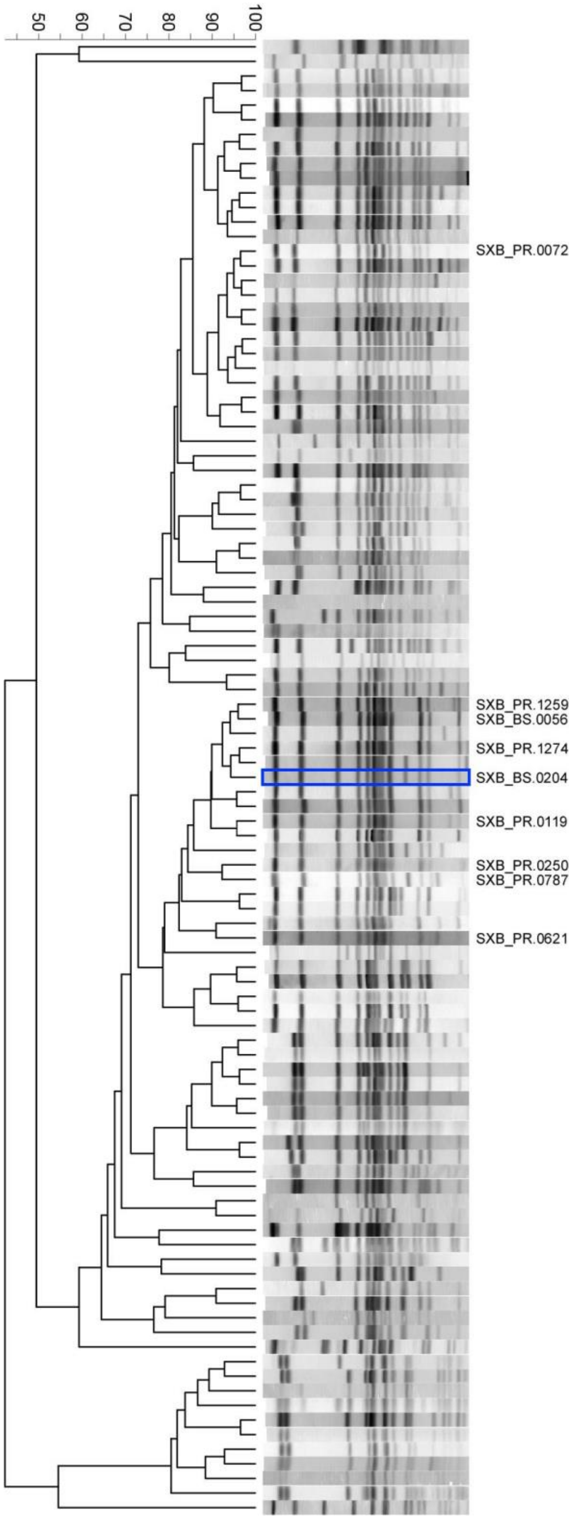

Supplementary Figure S1| UPGMA dendrogram of *S*. Derby PFGE profiles. PFGE types relevant to the study are reported. PFGE type SXB_BS.0204 is in the blue box.

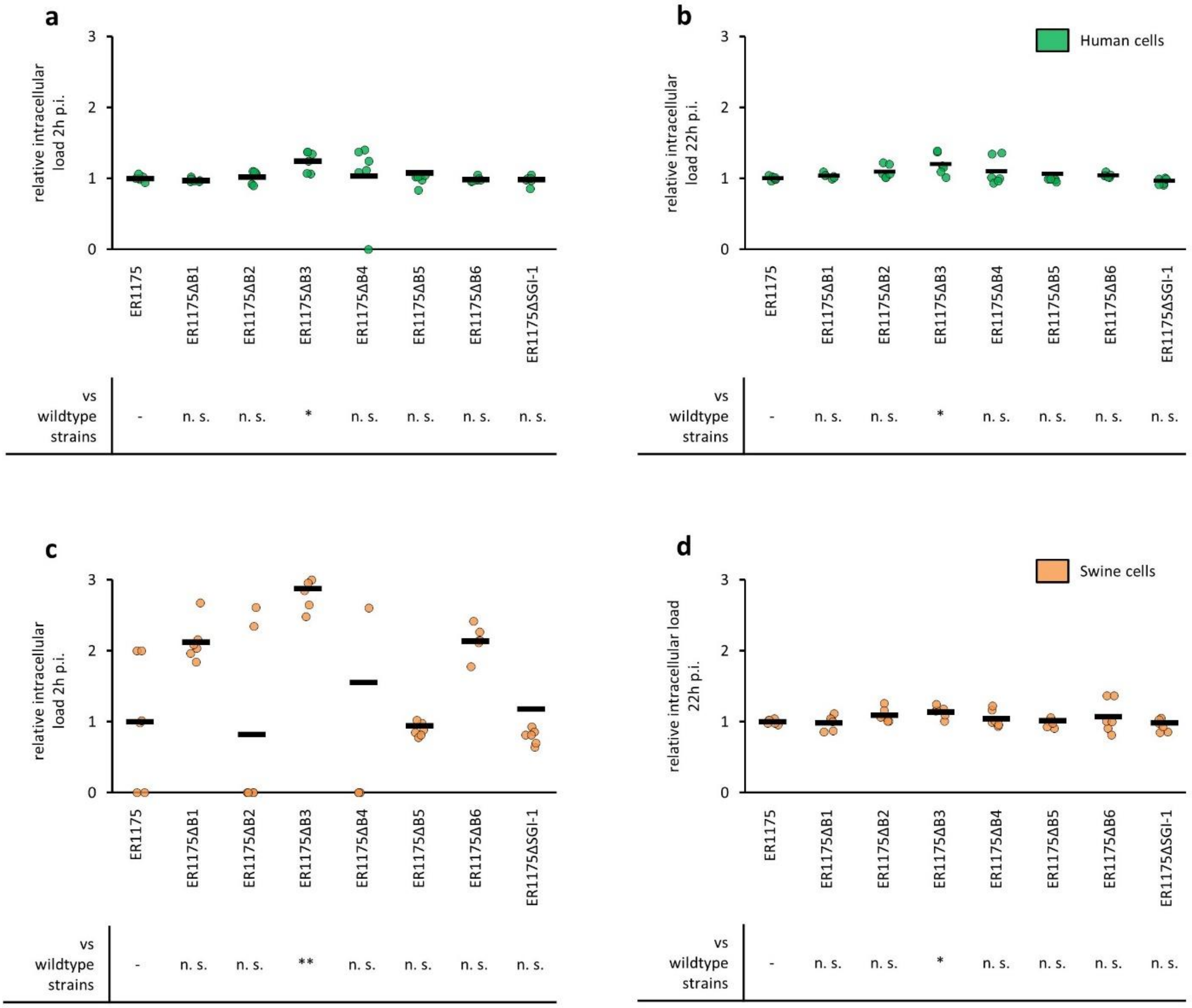

Supplementary Figure S2|Role of exclusive genes in virulence phenotype. Relative intracellular loads at 2h and 22h post infection (p.i.) of ER1175 and its mutants in human cells (**a** and **b**) and swine cells (**c** and **d**). Results are expressed as relative intracellular loads , i.e. the intracellular bacteria recovered after 2h and 22h of infection for each strain normalized on the respective wild type. Three biological replicates were performed and each tested in duplicate. Dots represent the values of the replicates. Arithmetic means are indicated by horizontal lines. Tables report p-values from two-tailed Student's t-test T test corrected for multiple comparisons (*p<0.05, **p<0.01, ***p<0.001, n.s. not significant).

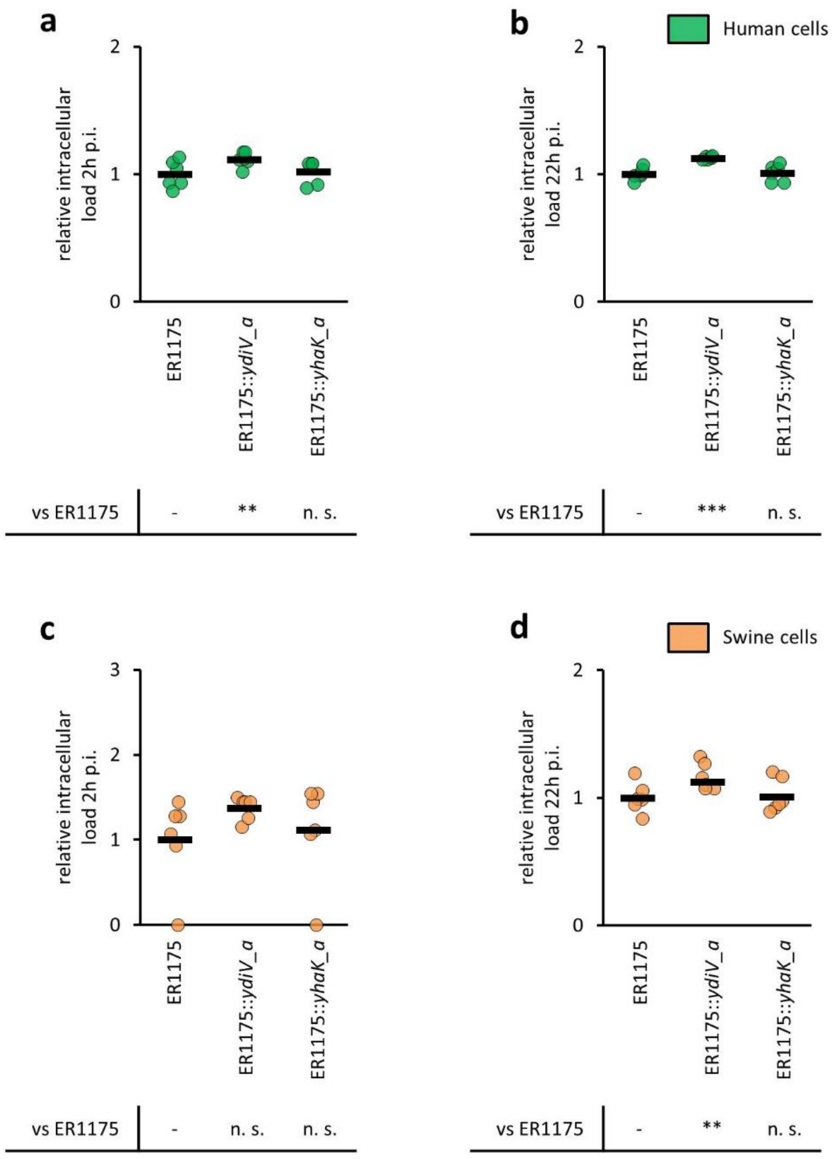

Supplementary Figure S3|Role of non-sense mutations in virulence phenotype. Intracellular loads at 2h and 22h post infection (p.i.) of ER1175, ER1175::*ydiV_a* and ER1175::*yhaK_a* in human cells (**a** and **b**) and swine cells (**c** and **d**). Results are expressed as relative intracellular loads, i.e. the intracellular bacteria recovered after 2h and 22h of infection for each strain normalized on the respective wild type. Three biological replicates were performed and each tested in duplicate. Dots represent the values of the replicates. Arithmetic means are indicated by horizontal lines. Tables report *p*-values from two-tailed Student's *t*-test corrected for multiple comparisons (**p*<0.05, ***p*<0.01, ****p*<0.001, n.s. not significant).

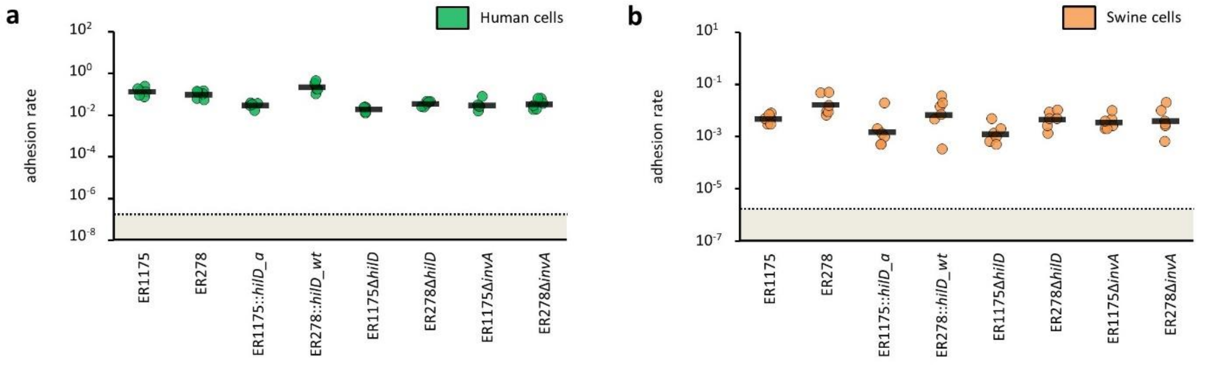

a

	ER1175	ER278	ER1175::*hilD_a*	ER278::*hilD_wt*	ER1175Δ*hilD*	ER278Δ*hilD*	ER1175Δ*invA*	ER278Δ*invA*
vs ER1175	-	n. s.	***	n. s.	***	**	**	*
vs ER278	n. s.	-	**	n. s.	***	**	*	n. s.

b

	ER1175	ER278	ER1175::*hilD_a*	ER278::*hilD_wt*	ER1175Δ*hilD*	ER278Δ*hilD*	ER1175Δ*invA*	ER278Δ*invA*
vs ER1175	-	n. s.	n. s.	n. s.	n. s.	n. s.	n. s.	n. s.
vs ER278	n. s.	-	n. s.	n. s.	**	n. s.	n. s.	n. s.

Supplementary Figure S4| The role of the *hilD* point mutation in the adhesion phenotype. Adhesion assays of ER1175, ER278 and their mutants in human cells (**a**) and swine cells (**b**). Results are expressed as adhesion rates, i.e. adhered bacteria recovered after 2h of infection divided by the inoculum. Three biological replicates were performed and each tested in duplicate. Dots represent the values of the replicates. Arithmetic means are indicated by horizontal lines. Tables report *p*-values from two-tailed Student's *t*-test corrected for multiple comparisons (*p<0.05, **p<0.01, ***p<0.001, n.s. not significant). The dotted line represents the limit of detection.

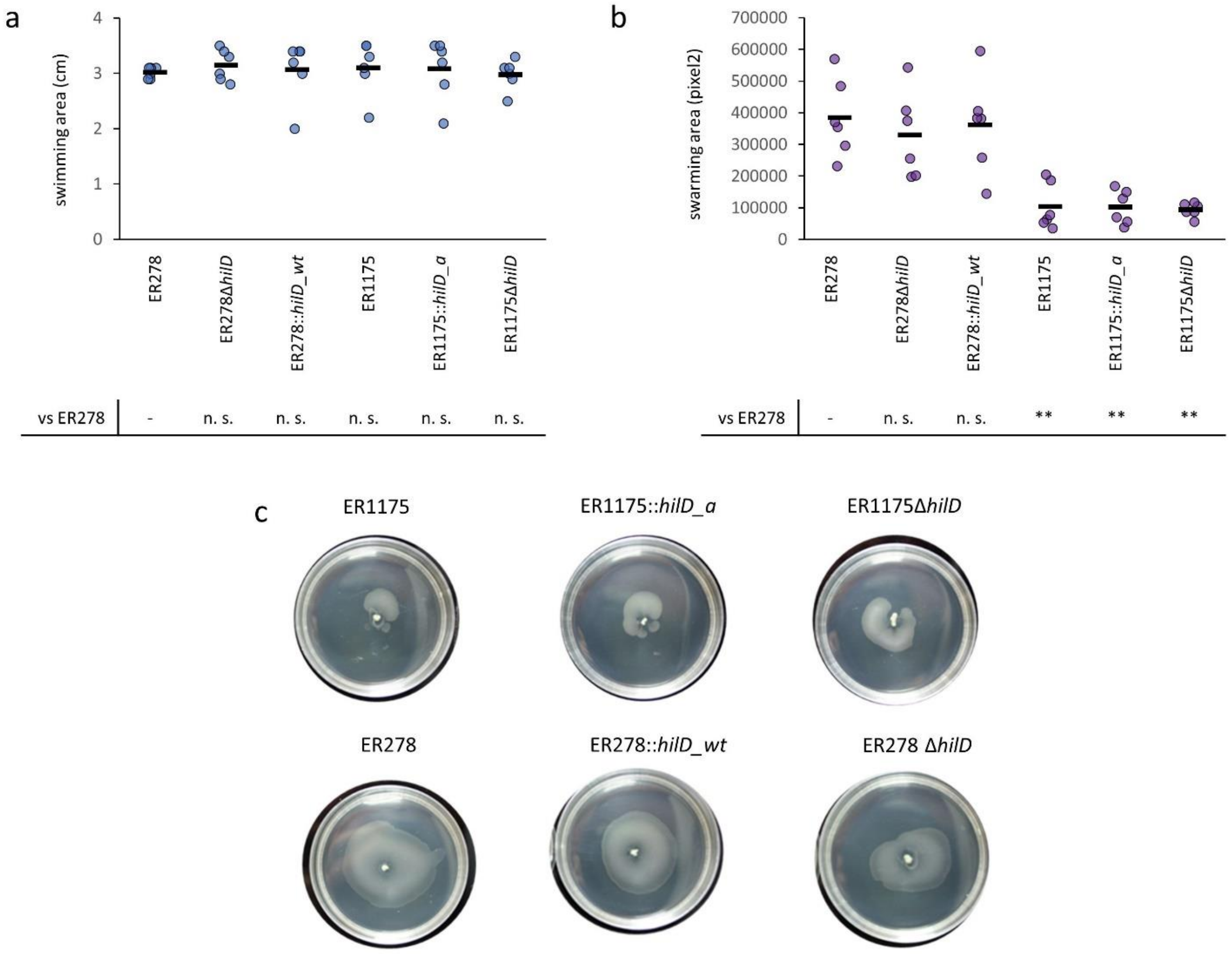

Supplementary Figure S5: **The role of HilD in motility phenotype.** Swimming motility was analysed on 0.3% agar plates after 6 hours incubation at 37°C. Swarming motility was analysed on plates containing 0.5% agar after 8 hours incubation at 37°C. Quantified swimming (a) and swarming (b) motility: dots represent the values of 3 biological replicates, each including 2 technical replicates. Arithmetic means are indicated by horizontal lines. Tables report *p*-values from two-tailed Student's *t*-test corrected for multiple comparisons (**p*<0.05, ***p*<0.01, ****p*<0.001, n.s. not significant). c) Representative swarming plates for each strain tested.

Supplementary Table 1| *Salmonella* serovar-host distribution in Emilia Romagna (Italy). The table reports: 1) total number (n°) of isolates and isolates from the major serovars by host and 2) percentage of each serovar in each host.

	**Human**		**Poultry**		**Cattle**		**Swine**	
**Serovars**	n°	Percentage	n°	Percentage	n°	Percentage	n°	Percentage
Total isolates	4373	100%	624	100%	174	100%	1164	100%
1,4,[5],12:i:-	2023	46.26%	17	2.72%	56	32,18%	430	36,94%
Typhimurium	610	13.95%	29	4.65%	49	28,16%	58	4,98%
Enteritidis	444	10.15%	52	8.33%	2	1,15%	11	0,95%
Napoli	148	3.38%	3	0.48%	1	0,57%	0	0,00%
Derby	120	2.74%	5	0.80%	6	3,45%	315	27,06%
Brandenburg	103	2.36%	0	0.00%	0	0,00%	37	3,18%
Infantis	99	2.26%	223	35.74%	6	3,45%	22	1,89%
Give	58	1.33%	4	0.64%	3	1,72%	21	1,80%
Panama	56	1.28%	0	0.00%	0	0,00%	4	0,34%
Rissen	43	0.98%	5	0.80%	5	2,87%	106	9,11%
Other serovars	669	15.30%	286	45.83%	46	26.44%	160	13.75%

Supplementary Table 4| Mutants generated in the study. CmR = Chloramphenicol resistance.

Strain	Relevant features
**ER1175ΔB1**	ΔB1::*cat*; CmR
**ER1175ΔB2**	ΔB2::*cat*; CmR
**ER1175ΔB3**	ΔB3::*cat*; CmR
**ER1175ΔB4**	ΔB4::*cat*; CmR
**ER1175ΔB5**	ΔB5::*cat*; CmR
**ER1175ΔB6**	ΔB6::*cat*; CmR
**ER1175ΔSGI1**	ΔSGI1::*cat*; CmR
**ER1175::*hilD_a***	Δ*hilD_wt*::*hilD_a-cat*; CmR
**ER1175Δ*hilD***	Δ*hilD_wt*::*cat*; CmR
**ER1175Δ*invA***	Δ*invA*::*cat*; CmR
**ER278::*hilD_wt***	Δ*hilD_a*::*hilD_wt-cat*; CmR
**ER278Δ*hilD***	Δ*hilD_a*::*cat*; CmR
**ER278Δ*invA***	Δ*invA*::*cat*; CmR

Supplementary methods

Construction of recombinant strains

For gene deletion, entire coding sequences (CDSs) were replaced with Chloramphenicol resistance cassette (*cat*) amplified from pKD3 template plasmid. Primers to amplify the recombining amplicon were designed with 50-nucleotide extension homologous to regions immediately up- and downstream of the CDS, and 20 nucleotides homologous to the *cat* resistance cassette. In allelic exchange experiments, linear fragments were constructed to contain the gene allelic variant and the *cat*-resistance cassette fused at the 3'. For *hilD*, the region including both the CDS and the 3'UTR sequence was used, because *hilD* 3'UTR is a cis-acting element directly involved in *hilD* mRNA turnover [1]. To obtain these constructs, the CDS or region of interest was amplified with the forward primer homologous to the 50-nucleotide sequence upstream the gene start codon and the reverse primer composed by 10 nucleotides homologous to the 5'end of the *cat*-resistance cassette and 10 nucleotides homologous to the 3' end of the CDS or region of interest. The *cat*-resistance cassette was amplified using the forward primer containing 10 nucleotides homologous to the 3' end of the CDS or region of interest and 10 nucleotides homologous to the 5'end of the *cat*-resistance cassette and a reverse primer designed with 50 nucleotides homologous to regions immediately downstream the CDS or region of interest and 20 nucleotides homologous to 3' end of the *cat*-resistance cassette. The two PCR products have therefore a 20bp overlapping region, designed to have an annealing T of ~60°C. The PCR products were assembled by Overlap PCR with the following cycle conditions, divided in two steps: for the first step 98°C 3 minutes, the annealing temperature of the overlapping region 2 minutes and 72°C 5 minutes, for the second step 98°C 30 seconds, then 98°C for 7 seconds, 62°C for20 seconds and 72°C for 1 minutes per 35 cycles, and 72°C for 7 minutes. In Overlap PCR, primers were added between the first and the second step. Competent cells were prepared from an overnight culture grown in 3 mL LB broth at 37°C with 200 rpm shaking. The culture was diluted 1:100 in LB broth and grown to OD_{600} = 0.6. One hour before reaching this OD_{600} value, L-arabinose (Sigma) was added to a final concentration of 10 mM to induce expression of recombinase genes. Once pelleted, cells were washed twice with ice-cold H_2O and

once with 10% glycerol. Cells were then suspended in 40 µL of 10% glycerol and mixed with DNA in a 0.1 cm electroporation cuvette (BioRad, Inc.). Cells were electroporated at 1.8 kV. Immediately after electroporation cells were let recover in SOC medium for 1–3 hours at either 30°C (for temperature-sensitive plasmids) or 37°C, with mild shaking (100 rpm). Cells were then concentrated ten-fold from a 1 ml culture and plated on LB agar containing Chloramphenicol (Cm) 20 µg/mL as selective media. Insertion of linear fragments was verified by PCR using two pairs of primers specific for the *cat*-resistance cassette and regions upstream and downstream of the target gene. For the allelic exchange, allelic-specific (AS) PCRs were then set up to detect the allelic variant carried by colonies positive to the previous PCR. A common reverse (or forward) primer and two forward (or reverse) AS primers were used for each SNP. Primers for AS-PCR were designed with BatchPrimer3 v1.0 [2] (https://wheat.pw.usda.gov/demos/BatchPrimer3/) or WASP [3] (https://bioinfo.biotec.or.th/WASP/home). The AS primers were designed with the 3' end complementary to each allele of the SNP. To guarantee specificity of allelic-specific primers one 'mismatch' at the second- or third-last base (at the 3'-end) of AS primers was added to improve the resulting AS PCRs.

Motility assay

To perform the swimming and swarming assays, bacteria were grown overnight at 37°C in LB broth in an orbital shaker (180rpm) and each culture was normalized to an optical density value (OD_{600}) of 1. Swimming and swarming motility were assayed on freshly prepared LB plates with D-Glucose 12mM, containing 0.3% [4] and 0.6% [5] bacteriological agar, respectively. Plates were inoculated by poking the agar with a sterile needle previously soaked into the normalized bacterial cultures. Plates were incubated at 37°C and swimming and swarming motility were analysed 6h and 8h post incubation, respectively. The swimming motility was assessed by measuring the diameters of the swimming halos. The area of the swarming halos was measured in pixel using AxioCam ICm1 ZEN2012 Blue Edition. The bacterial growth area was measured in three independent experiments, with each experiment performed in duplicate. Statistical significance was determined using a Student's *t*-test.

Gene expression analysis during epithelial cell infection

For gene expression analysis during epithelial cell infection the protocol described for cell culture infection assays was used with some modifications. Treatment with gentamycin was removed to collect both adherent and intracellular bacteria. 10^7 INT-407 cells were seeded on a 75 cm^2 tissue culture flask and incubated 48 hours at 37°C and under 5% CO^2. The overnight bacterial cultures were diluted in DMEM containing 10% fetal bovine serum and used to inoculate the epithelial cell monolayer using a MOI 10. RNA was extracted at 0 (t0), 30 (t30) and 60 (t60) minutes post-inoculation. For t0, each bacterial culture was diluted to a MOI of 10 and RNA was extracted without inoculation on epithelial cells. At 30 and 60 minutes of infection the flasks were washed 5 times with PBS and eventually 5 ml of ice cold 'eukaryotic cells lysis and RNA stabilization solution' (0.1% SDS, 19% ethanol, 1% acidic phenol in water [6]) were added. The flasks were incubated on ice for 30 minutes, then intracellular and adherent *Salmonella* were collected by centrifugation (27,500g for 10 minutes at 4°C). RNA was extracted from three biological replicates for each strain by RNeasy Mini Kit. RNase-Free DNase Set was used to digest trace DNA potentially contaminating the sample. High-Capacity cDNA Reverse Transcription Kit was used to generate complementary DNA (cDNA). The expression of 8 virulence genes was analyzed by qRT-PCR. Each reaction was performed in two technical replicates for each sample. Expression of target genes were normalized to the reference gene *gmk*. ER1175-t0 was used as internal control condition. The relative transcriptional levels were calculated by the $2^{-\Delta\Delta Ct}$ method corrected for exact PCR efficiencies. Differences in gene expression among strains were analyzed using unpaired *t* test. Bonferroni correction was applied for multiple comparisons.

Supplementary References

1. Lopez-Garrido, J., Puerta-Fernandez, E. & Casadesus, J. A eukaryotic-like 3 untranslated region in *Salmonella enterica* hilD mRNA. *Nucleic Acids Res.* **42(9),** 5894–5906 (2014).

2. You, F. M. *et al*. BatchPrimer3: a high throughput web application for PCR and sequencing primer design. *BMC Bioinformatics* **9,** 253 (2008).

3. Wangkumhang, P. *et al*. WASP: a Web-based Allele-Specific PCR assay designing tool for detecting SNPs and mutations. *BMC Genomics* **8,** 275-283 (2007).

4. Kearns, D. B. A field guide to bacterial swarming motility. *Nat. Rev. Microbiol.* **8(9),** 634-44 (2010).

5. Kim, W. & Surette, M. G. Prevalence of surface swarming behavior in *Salmonella*. *J. Bacteriol.* **187(18),** 6580-3 (2005).

6. Hautefort, I. *et al*. During infection of epithelial cells *Salmonella enterica* serovar Typhimurium undergoes a time-dependent transcriptional adaptation that results in simultaneous expression of three type 3 secretion systems. *Cell. Microbiol*. **10,** 958–984 (2008).
